# Sick sinus syndrome associated with anti-programmed cell death-1

**DOI:** 10.1186/s40425-018-0388-9

**Published:** 2018-07-16

**Authors:** Chien-Yi Hsu, Yu-Wen Su, San-Chi Chen

**Affiliations:** 1 Institute of Clinical Medicine, National Yang-Ming University, Taipei, Taiwan; 2 Cardiovascular Research Center, National Yang-Ming University, Taipei, Taiwan; 3 Department of Internal Medicine, School of Medicine, College of Medicine, Taipei Medical University, Taipei, Taiwan; 4 Division of Cardiology and Cardiovascular Research Center, Department of Internal Medicine, Taipei Medical University Hospital, Taipei, Taiwan; 5 Division of Endocrinology and Metabolism, Department of Medicine, Taipei Veterans General Hospital, Taipei, Taiwan; 6 Division of Medical Oncology, Center for Immuno-oncology, Department of Oncology, Taipei Veterans General Hospital, No. 201, Sec. 2, Shipai Road, Taipei, Taiwan 11217; 7 Faculty of Medicine, National Yang-Ming University, Taipei, Taiwan

**Keywords:** Sick sinus syndrome, Adrenal insufficiency, Anti-programmed cell death-1, Immune-related adverse event, Hepatocellular carcinoma

## Abstract

**Background:**

Use of anti-programmed cell death-1 (anti-PD-1) has been successful in treating many types of cancers. Despite its promising efficacy, immune-related adverse events are still a major concern. Immune-related cardiotoxicity, which is rare but fatal, has recently become a focus of attention. Cardiotoxicities including myocarditis, cardiomyopathy, cardiac fibrosis, heart block and cardiac arrest have been reported. Of these toxicities, myocarditis is often accompanied by dysrhythmia. The presentation of sick sinus syndrome as an immune-related adverse event has not yet been reported. Here, we reported the first case of sick sinus syndrome, a rare toxicity induced by anti-PD-1.

**Case presentation:**

A 42-year-old male patient who had metastatic hepatocellular carcinoma failed treatment with sorafenib. Pembrolizumab at a fixed dose of 100 mg every three weeks was given. His heart rate gradually slowed down and he presented sick sinus syndrome with a lowest heart rate of 38 bpm after six cycles of pembrolizumab. He denied chest tightness, cold sweating, palpitation and dyspnea. Lab data including cardiac enzyme, electrolytes and thyroid function were all within a normal range. Simultaneously, he complained of fatigue, dizziness and anorexia with hypotension. Lab data revealed low cortisol and ACTH levels. Anti-PD-1 induced adrenal insufficiency was suspected. Low-dose cortisone (12.5 mg) was prescribed, and the patient’s symptoms, hypotension and sick sinus syndrome showed rapid improvement. Cortisone was gradually titrated and discontinued three weeks later. His sick sinus syndrome did not relapse and the cortisol and ACTH level returned to normal.

**Conclusions:**

Sick sinus syndrome caused by anti-PD-1 treatment is a rare adverse event. With the development of sick sinus syndrome, myocarditis should be the first differential diagnosis because of its lethality. From this case, we learned that sick sinus syndrome may be a presentation of immune- or adrenal insufficiency-mediated sinus node dysfunction, both could be reversed with a glucocorticoid supplement.

## Background

Immune checkpoint inhibitors targeting cytotoxic T-lymphocyte-associated antigen-4, and programmed cell death protein (PD-1) are emerging as forceful weapons against various tumors, including melanoma, renal cell carcinoma, lung cancer, and bladder cancer. However, due to the immunomodulatory nature of immune checkpoint inhibitors, a new spectrum of immune-related adverse events (irAE) associated with checkpoint inhibitors, anti-CTLA4 antibody and anti-PD-1 antibody has been recognized [1].

Some types of irAE, such as pneumonitis, hepatitis, and cardiac toxicities, are life-threatening and need to be diagnosed immediately. Among these irAE, immune-related cardiac toxicities have been highlighted recently [2]. Dysrhythmia, one form of cardiac toxicity, may present with myocarditis. However, sick sinus syndrome following anti-PD-1 treatment has not been reported.

The endocrine organs typically affected by irAE are the pituitary, thyroid and adrenal glands, and the manifestations are hypophysitis, isolated secondary adrenal insufficiency, hyper−/hypothyroidism and primary adrenal insufficiency [3]. Overall incidence of irAE is approximately 10%, but this varies among different endocrinopathies and treatment agents [4]. The irAE of adrenal insufficiency typically presents with fatigue and hypotension, but rarely with dysrhythmia. Here, we present the first reported case of sick sinus syndrome associated with anti-PD-1 treatment.

## Case presentation

This 42-year-old male patient was a HBV carrier with regular follow-up. He had no symptoms, but abdominal sonography in a routine examination revealed a liver tumor. His blood pressure was 123/86 mmHg, and heart rate (HR) 84 bpm. Laboratory data revealed AST/ALT = 58/58 U/L, total bilirubin = 1.2 mg/dL, albumin = 4.3 g/dL, HBV DNA titer 37,600 copies, and AFP level = 121 ng/mL. Abdominal computed tomography (CT) showed a large tumor (13.0X7.0 cm) and several adjacent small nodules with typical arterial enhancement and portovenous washout. The patient was diagnosed with hepatocellular carcinoma (HCC), Barcelona clinic liver cancer (BCLC) stage B, and then underwent extensive left lobectomy. The pathology report revealed metastatic lymphadenopathy; thus, his cancer stage was revised to BCLC stage C.

This patient started treatment with sorafenib 400 mg twice per day for his metastatic HCC. However, grade 3 hand-foot syndrome developed, and then sorafenib was gradually titrated to 200 mg once daily. Two months after initiation of sorafenib, his AFP level increased from 121 to 1152 ng/dL, and follow-up CT scan showed an increase in the size of the intra-abdominal lymph nodes; therefore, progressive disease was confirmed. With a thorough evaluation and having obtained the patient’s informed consent, an off-label treatment with pembrolizumab at a reduced dose of 100 mg (due to his financial situations) every three weeks was administered.

**Fig. 1:**
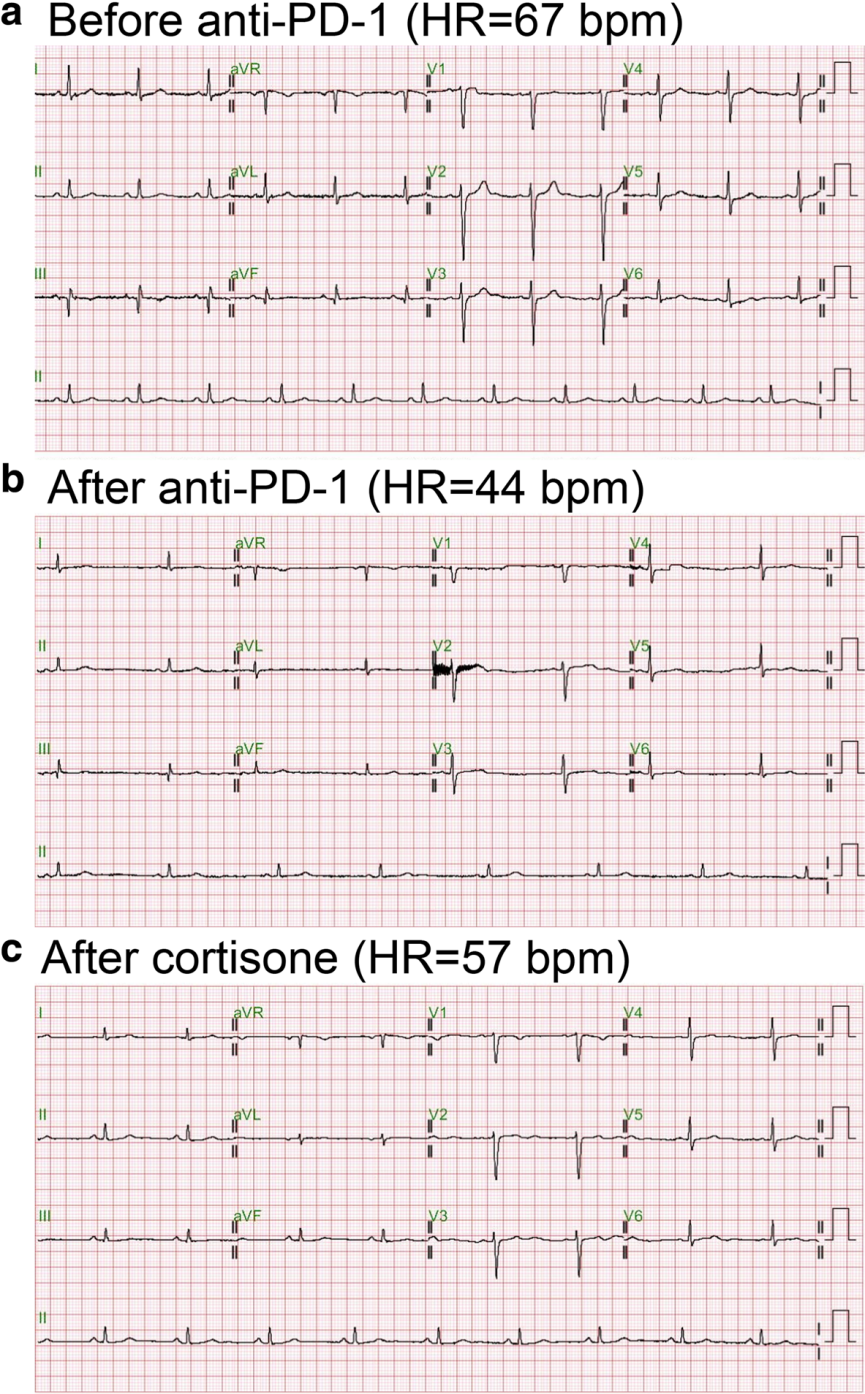
Electrocardiography (ECG) results. **a.** Baseline ECG. **b.** Sick sinus syndrome developed after anti-PD-1 treatment. **c.** Sick sinus syndrome was reversed after cortisone treatment

**Fig. 2:**
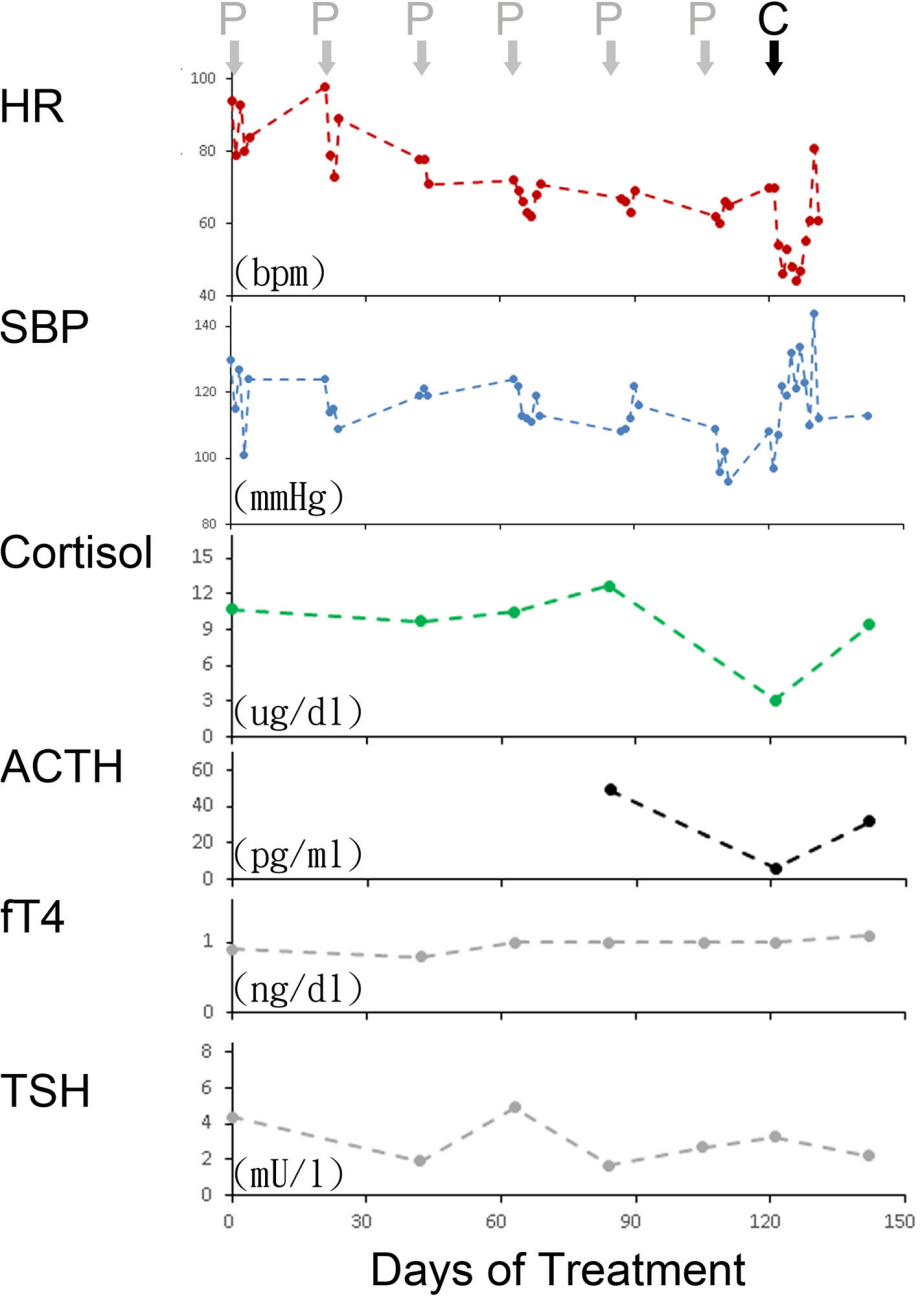
Serial vital signs and hormone levels following anti-PD-1 treatment. Heart rate (HR) and systolic blood pressure (SBP) dropped after six cycles of pembrolizumab (P) treatment. Cortisol and adrenocorticotropic hormone (ACTH) levels declined, but thyroid-stimulating hormone (TSH) and free T4 (fT4) remained within a normal range. HR and SBP were reversed after prescription of cortisone. Cortisol and ACTH levels returned to a normal range about one month later

The patient did not experience an irAE until after six cycles of pembrolizumab had been prescribed. Grade 2 fatigue, dizziness and anorexia were complained. The systolic blood pressure declined to 90 mmHg. After fluid resuscitation, his symptoms and hypotension were partially improved. However, his HR dropped with the slowest 38 bpm few days later. He denied chest tightness/pain, cold sweating, palpitation and dyspnea. Physical examination disclosed regular and slow heart beats without murmur, no engorged jugular vein and no leg edema. Electrocardiography (ECG) showed sinus bradycardia (Fig. 1b). Laboratory data revealed CPK = 19 U/l, Troponin-*I* < 0.4 ng/ml, Na = 140 mEq/L, K = 4.6 mEq/L, Ca = 8.1 mg/dl and Mg = 1.9 mg/dl that were all within a normal range. A cardiologist was consulted for the symptomatic bradycardia. The 24-h Holter Monitoring was performed which revealed marked sinus bradycardia (minimal heart rate < 40 bpm at day time) with intermittent atrial and ventricular premature contractions. There was no long pause more than 2 s on Holter, but chronotropic incompetence with easily fatigue and light-headedness during moderate physical activity was noted. Cortisol level maintained in the normal range in regular check-up, but dropped to 3.1 (3.7–19.4) mcg/dl after six cycles of pembrolizumab. ACTH level declined to 6.4 (7–46) pg/ml at the same time (Fig. 2).

Backtracking his medical records, his HR gradually slowed down while the systolic pressure and cortisol level remained normal. Both immune-mediated sinoatrial node dysfunction and adrenal insufficiency were suspected. Since systemic steroids could treat both irAEs, a low dose of cortisone (12.5 mg) was prescribed orally once daily. His symptoms, bradycardia and hypotension improved immediately after one dose of cortisone. Three weeks later, cortisone was discontinued without symptoms relapse. Pembrolizumab was also discontinued due to the progressive status of his HCC.

## Discussion and conclusions

Immune checkpoint inhibito r therapy can induce a spectrum of cardiac side effects, including autoimmune myocarditis, cardiomyopathy, cardiac fibrosis, heart block and cardiac arrest, although these are rare events [5]. There are several possible mechanisms for the observed cardiotoxic effects. T-cells could target an antigen shared by the tumor and the heart muscle [6]. In animal studies, PD-1 played a role in myocardial immune responses and protected against inflammation and myocyte damage in mice models of T-cell–mediated myocarditis [7].

Regarding the cardiac pro-arrhythmic effects caused by checkpoint inhibitor therapy, Johnson et al. recently reported a case of complete heart block and cardiac arrest following initial doses of nivolumab and ipilimumab [6]. The adverse effects of sinus node dysfunction induced by checkpoint inhibitors have not been reported previously. However, elevated levels of proinflammatory markers, such as interleukin-6 and C-reactive protein, increase the risk of supraventricular and ventricular cardiac arrhythmias and their complications [8, 9]. Moreover, inter-atrial block frequently coexists with sinus node disease and is considered to be caused by immune-related myocardial inflammation or during the development of heart failure [10]. Our report raises the concern of a potential adverse effect of sinus node dysfunction during immune checkpoint inhibitor therapy. Physicians treating patients with checkpoint inhibitors should be aware of their potentially cardiotoxic effects, especially in patients with preexisting cardiac conditions. These high-risk patients should be closely monitored for deterioration of heart function. The timely and prompt management of cardiac side effects, including administration of systemic steroids, is important to avoid a potentially poor prognosis or fatal outcome [11].

Pembrolizumab induced adrenal insufficiency has been reported [12]. Adrenal insufficiency, either primary or secondary, leads to clinical presentations of fatigue, anorexia, nausea, fever, abdominal pain, low blood pressure, and hypoglycemia [13]. Although less common than thyroid diseases, isolated adrenal insufficiency may also induce ECG changes. Associations between adrenal insufficiency and T wave inversion, QT prolongation, and even torsades de pointes have been reported [14–17]. The underlying pathophysiology remains uncertain. Current evidence from animal studies showed that glucocorticoid probably influences the expression of potassium channels, for instance, I_Ks_ (mink, KvLQT1) and I_Kr_ (hERG, MiRP1), within ventricles, via serum- and glucocorticoid-inducible kinase (SGK1) [18, 19]. Another study suggested that glucocorticoid helps maintain calcium transport in the sarcoplasmic membrane, and that adrenal insufficiency with calcium overload might be related to QT prolongation [20].

Two important differential diagnosis of this patient include immune-mediated and adrenal insufficiency-induced sinus node dysfunction. The limitation is that it would be difficult to prove the autoimmunity of sinus node induced by anti-PD-1 treatment. Tracing his medical records, his sinus bradycardia gradually progressed, whereas hypotension and low cortisol level did not developed until six cycles of pembrolizumab. Hence, immune- mediated sick sinus syndrome may be more likely. On the other hand, ACTH stimulation test that is useful to establish the diagnosis of adrenal insufficiency was not performed in this case.

Sick sinus syndrome is a rare toxicity caused by anti-PD-1. Cardiac toxicities such as myocarditis should always be considered first, due to the fatal outcome. Endocrine disorders including dysfunction of the pituitary, thyroid and adrenal glands should also be differentiated. Steroids could be the treatment choice based on the experience from this case.

## Abbreviations

BCLC: Barcelona clinic liver cancer
ECG: Electrocardiography
HCC: Hepatocellular carcinoma
irAE: Immune-related adverse event
PD-1: Programmed cell death protein-1
